# ABO GENE AND AGING-RELATED OUTCOMES IN OVER 379,000 EUROPEAN-DESCENT PARTICIPANTS OF UK BIOBANK

**DOI:** 10.1093/geroni/igz038.3369

**Published:** 2019-11-08

**Authors:** Chia-Ling Kuo, Ziwei Pan, Luke C Pilling, George A Kuchel, David Melzer

**Affiliations:** 1 University of Connecticut, Farmington, Connecticut, United States; 2 University of Connecticut Health, Farmington, Connecticut, United States; 3 University of Exeter, Exeter, United Kingdom; 4 University of Connecticut School of Medicine, Farmington, Connecticut, United States; 5 University of Exeter, Exeter, Devon, United Kingdom

## Abstract

Genetic variants associated with multiple traits are potential targets to delay aging. Drugs supported by genetic evidence are twice as likely to succeed in human trials. Single nucleotide polymorphisms (SNPs) in the ABO gene were reported by genome-wide associations studies, associated with breast cancer, coronary artery disease, stroke, and type II diabetes. To evaluate the potential of ABO gene as a target for aging intervention, we conducted a phenome-wide association study (PheWAS) to associate the genotype-derived blood types (based on two SNPs in the ABO gene) with a wide range of aging-related outcomes. The genotype-derived blood type distribution (41% A, 9% B, 3% AB, and 47% O) is similar to that reported by the UK National Health Service (39% A, 10% B, 3% AB, and 48% O). The blood type was not associated with parental lifespan or extreme parental longevity. Non-O types had modestly lower risk of hypertension than O type but higher risk of type II diabetes and pancreatic cancer (e.g., OR= 1.37, 95% CI: 1.17 to 1.59 comparing A to O). Additionally, “A" type had modestly higher risk of breast cancer than other types. “A” allele (in A or AB type) was associated with lower heel bone mineral density, alkaline phosphatase (e.g., 0.41 standard deviation lower in A than that in O, 95% CI: -0.42 to -0.40), and hemoglobin concentration, but higher HbA1c, direct LDL, and cholesterol. Blood types with A allele(s) are less favored than other blood types, which however are adversely associated with some aging traits.

